# UNIQUE TRANSKINGDOM MICROBIOME SIGNATURES LINKED WITH COGNITIVE DECLINE IN OLDER ADULTS OF MIAGB CONSORTIUM COHORT

**DOI:** 10.1093/geroni/igac059.2781

**Published:** 2022-12-20

**Authors:** Diptaraj Chaudhari, Shalini Jain, Hariom Yadav, Michal Masternak, Peter Holland, Marc Agronin, MiaGB Consortium Team

**Affiliations:** University of South Florida, Tampa, Florida, United States; University of South Florida, Tampa, Florida, United States; University of South Florida, Tampa, Florida, United States; University of Central Florida, Orlando, Florida, United States; Florida Atlantic University, Boca Raton, Florida, United States; Miami Jewish Health, Miami, Florida, United States; University of South Florida, Tampa, Florida, United States

## Abstract

The prevalence of age-related cognitive disorders is increasing. Effective prevention and treatment interventions are unavailable due to a poor understanding of aging biology. Multiple emerging evidence indicates that the gut microbiome is linked with age-related disorders; however, their clinical importance in differentiating and predicting the risk of cognitive decline or dementia is largely elusive. Utilizing samples and data of a large, multi-site clinical study across the state of Florida called Microbiome in aging Gut and Brain (MiaGB) Consortium, our whole genome microbiome sequencing revealed that the viral and archaeal population was significantly reduced in the gut of older adults with dementia (n=8) compared to those with mild cognitive impairment (MCI) (n=25) and normal cognition (n=59). Whereas the fungi were exclusively detected in the controls only. Alpha diversity of the participants with MCI and dementia was lower than the cognitively healthy controls. The abundance of Actinobacteria and Verrucomicrobia phyla was higher, and Firmicutes phylum was lower in the participants with dementia. Bacteriophages Lactobacillus prophage Lj771 and Microbacterium phage Min1 were exclusively detected in the gut of the participants with dementia. The study also identifies key metabolic pathways altered in the controls versus the cognitive impairment state. Our biomarker discovery analyses also revealed that these unique microbiome signatures and pathways might have predictive power for cognitive decline and dementia risk and offer new targets for future therapeutic interventions.

